# Gene expression and morphological responses of *Lolium perenne* L. exposed to cadmium (Cd^2+^) and mercury (Hg^2+^)

**DOI:** 10.1038/s41598-021-90826-y

**Published:** 2021-05-27

**Authors:** Yuby Cruz, Sharik Villar, Karen Gutiérrez, Carolina Montoya-Ruiz, Jorge L. Gallego, Maria del Pilar Delgado, Juan F. Saldarriaga

**Affiliations:** 1 Department Civil and Environmental Engineering, Universidad de los Andes, Carrera 1Este #19A-40, Bogotá, Colombia; 2 Department Biological Sciences, Universidad de los Andes, Carrera 1 #18A-10, Bogotá, Colombia; 3 Facultad de Ciencias, Universidad Nacional de Colombia, Sede Medellín Calle 59A #63-20, Medellín, Colombia 050034; 4 Environmental Research Group (GIA), Department Engineering, Fundación Universitaria Tecnológico Comfenalco, Carrera 44 D # 30A-91, 130015 Cartagena, Colombia

**Keywords:** Biotechnology, Molecular biology, Plant sciences, Environmental sciences, Solid Earth sciences

## Abstract

Soil contamination with heavy metals is a major problem worldwide, due to the increasing impact mainly caused by anthropogenic activities. This research evaluated the phytoremediation capacity of, *Lolium perenne* for heavy metals such as cadmium (Cd^2+^) and mercury (Hg^2+^), and the effects of these metals on morphology, biomass production, and the changes on gene expression. Seeds of *L. perenne* were exposed to six concentrations of Cd^2+^ and Hg^2+^ in the range of 0 to 25 mg L^−1^, and two mixtures of Cd^2+^–Hg^2^. The Non-Observed Effect Level (NOEL) was established with dose response curves and the expression of specific genes was evaluated applying a commercially available quantitative reverse transcription (RT-qPCR) assay. There was no significant effect when exposing the seeds to Hg^2+^, for Cd^2+^ the maximum concentration was established in 0.1 mg L^−1^, and for the two concentrations of mixtures, there was a negative effect. An increase of expression of genes that regulate antioxidant activity and stress was found when the plant was exposed to heavy metals. Given the high tolerance to metals analyzed that was reflected both, the development of the plant and in its molecular response, these results highlight that *L. perenne* is a plant with phytoremediator potential.

## Introduction

Soil contamination with heavy metals has become an important environmental problem worldwide, because of their high toxicity for the ecosystems and human health, even in small doses^1–3^. The increase of heavy metal contamination is mainly caused by anthropogenic activities such as mining, agriculture, industrialization, and urbanization^4–7^. In particular, this is a critical issue on developing countries, where the need of economic growth overtake the rules of environmental protection, being the priority to look strategies to repair or mitigate the environmental damage^2^.

Cadmium (Cd) and mercury (Hg) are the principal heavy metals that have been increasing their concentration in soils due to anthropogenic activities^8^. It is estimated that the natural concentration of cadmium in soils is between 0.1 and 1.0 mg/kg while the highest concentration is around 10 mg/kg. While for mercury, concentrations between 0.11 and 36.9 mg/kg have been reported in mining soils in Colombia^9,10^. The increasing of inorganic cadmium (Cd^2+^) in soils is mainly generated by the used of phosphate fertilizers in the agriculture and the oil refinery^11,12^.The increase of mercury ions (Hg^2+^) is generated by the amalgamation of gold typically used in illegal mining^13^. Aimed to reduce the concentration of heavy metals in soils, diverse strategies have been implemented based on chemical, physical and biological processes^14^. The phytoremediation is one the biological process that reduces the concentrations of heavy metals through the growing of plants, which absorb, transfer, or transform the metal, this process is considered cost-effective and environmentally friendly^14,15^.

Within the phytoremediator plants, *Lolium perenne* (ryegrass) is a bunchgrass native of temperate regions of Europe, Asia, and North Africa, and it is widely distributed throughout the world, including America, and Australia^16,17^. The responses of this plant when exposed to abiotic stress are known at the morphological and cellular level. However, information on the effects in relation to dose-time and molecular activity is scarce^18–20^. Regarding the morphological response, a promising result has been found in the plant characterized by rapid germination, root development, sensitivity to metals (no mercury transformation), resistance to environmental stress, and wide distribution^18,19,21^. In contrast, information is scarce on the adaptation mechanisms of *L. perenne*. This information is necessary, because one of the first signs of adverse effects that occur in organisms exposed to stress conditions is an alteration in gene expression that reflects physiological changes to adapt to new unfavorable conditions^22^.

It has been demonstrated that *L. perenne* can tolerate high salinity conditions through molecular interactions involving genes related to the enzymatic antioxidant system, activation of shock thermic stress proteins, mitochondrial metabolism, among others^23^. The activation of the enzymatic antioxidant system in the cell is one of the molecular mechanisms during the plant exposure to heavy metals. However, the production of reactive oxygen species (ROS) increases, and could lead lipid peroxidation, membrane damages, and enzyme inactivation ^4^. This system involves different enzymes such as catalase (CAT), peroxisomal ascorbate peroxidase (pAPX*)*, glutathione reductase (GR), glutathione S-transferase (GST), glutathione peroxidase (GPX), superoxide reductase (SOD), and non-enzymatic substances production as ascorbic acid, α-tocopherol, and carotenoids^24^.

Most of the early responses to metal toxicity are mediated by the induction of heat shock proteins as Hsp70 and Hsp90, which are chaperons that are activated in any stress condition to promote the homeostasis in plant cells^25^. Hsp90 protein are responsible for safeguarding the integrity of the cell membrane when a plant organism is exposed to heavy metals, resulting in reductions in the rate of root germination and length^26^. Hsp70 perform the catalysis necessary to recruit and release proteins quickly^26,27^.

On the other hand, the hormetic-growth could be due by effects in photosynthesis and respiration routes. Light-harvesting chlorophyll a/b-binding protein (Lhcb1), serves as a complex antenna, transferring the energy obtained from the absorption of sunlight to photosystem II, to boost electron transport in photosynthesis. Succinate dehydrogenase flavoprotein subunit-mitochondrial precursor (SDH) is an essential part of the Krebs cycle and plant respiration. Previous evidences show that the genes that encodes for these proteins has been modulated during *L. perenne* growth on high salinity soil conditions^23^.

According to the lack of knowledge about the mechanisms used by *L. perenne* to grow in soil contaminated with heavy metals, the few information about growth during the time, and the necessity to find environmentally friendly strategies to remediated soil that has been contaminated with metals like cadmium and mercury. This study evaluated the phytoremediation capacity of *L. perenne* for Cd^2+^ and Hg^2+^ contamination, the germination kinetics and growth, and the changes in the expression of the genes associated to the enzymatic antioxidant system, activation of shock thermic stress protein, and mitochondrial metabolism.

## Experimental

### Growth of *Lolium perenne*

*Lolium perenne* seeds reference Rye Grass Bestfort plus (X CEBA, Bogotá, Colombia) were washed with deionized water and 10% KOH placed in Petri dishes (10 cm in diameter and 2.5 cm deep) with Whatman 0.2 mm filter paper. Each Petri dish was watered with 7 ml of Cd^2+^ or Hg^2+^ solution at concentrations of 0.05, 0.10, 0.25, 1.00, 5.00 and 25.00, which were selected according to previous studies and the actual concentrations reported in contaminated soils^28,29^. Binary mixtures of Cd^2+^–Hg^2+^ were also evaluated at concentrations of 0.1 and 0.25 mg/L randomly selected within the range previously reported^28,29^. The Cd^2+^ solution was prepared by diluting CdCl_2_-2.5H_2_O (Alfa Aesar, Hampton, USA) in deionized water, and the Hg^2+^ solution diluting HgCl_2_ (Sigma-Aldrich, San Luis, USA). The seeds were exposed in a 12 h/12 h photoperiod with temperatures of 20/16 ºC (light/day) and relative humidity of 52%. The cultures were observed daily and if necessary were moistened with 3 ml of the corresponding solution. Seed boxes watered with distilled water was included as negative controls. According to Decree 309 of 2000 in Colombia, study permits are not necessary if collections or captures will not be carried out, and the research has focused on the sowing of seeds acquired by the researchers.

### Determination of morphological changes

#### Germination

For the germination test, 60 seeds were added to each Petri dish, and they were grown as mentioned above. The number of germinated seeds were counted daily for each of the concentrations of Cd^2+^, Hg^2+^ and Cd^2+^ + Hg^2+^ until day nine, time when the maximum percentage of germination was achieved, this time was determined by doing a preliminary germination test with the control. Four replicates of the culture were made for each treatment and for the control. With the obtained data, the final germination percentage (FGP), initial germination percentage (IGP), the germination speed coefficient (CVG), the average germination time (MGT), and the germination rate index (TGI) was established according to the methodology proposed by Al-Ansari and Ksiksi^30^.

#### Elongation parameters

To evaluate the effect on elongation, the cultures were made with the same conditions and replicates as in the germination test. Roots elongation was measured daily^31^ by photography register using the software Image J (NIH, USA). On the surface of each of the Petri dishes, a 5 × 5 grid was marked, forming 25 squares of 1 cm, in each of these squares a seed was placed. To evaluated elongation at the end point, on day 9 after seed sowing the length of the root and stem were measured using a PCE-DCP 600 N Vernier caliper (HOPEX, Colombia) with an accuracy of 0.01 mm.

### Evaluation of gene expression

*Lolium perenne* seeds were grown in concentrations of 0.05 and 0.1 mg/L of Cd^2+^ and Hg^2+^ and 0.1 and 0.25 mg/L for the binary mixture, which correspond to the toxicity threshold (NOEL). Also, a negative control without contaminant was grown. On day 9 of sowing the seeds, roots and stems were cut using sterile scalpels and were collected in sterile 1.5 mL Eppendorf tubes to be stored at − 80 °C until the subsequent RNA isolation. For this procedure approximately 50–150 mg of tissue were used, it was macerated using liquid nitrogen and then processed using Agilent Plant RNA Isolation Mini Kit (Agilent Technologies, USA), according to the manufacturer's recommendations.

Isolated RNA was quantified using Nanodrop and its integrity confirmed by a 1.5% agarose gel stained with GelRed (Biotium, Fremont, USA). The extracted RNA was treated with DNAse (Thermo scientific, Waltham, USA) and each sample was diluted to a concentration of 30 ng/μl for processing. This assay was performed by duplicate for each treatment concentrations and the negative control.

The genes evaluated were selected according to the previously reported sequences and their evidence in their participation in tolerance processes of the plant response to growth in contaminated soils^23^. These genes encode for proteins involved in detoxification routes: *CAT* gene coding for catalase, *pAPX* gene for enzymatic ascorbate peroxidase, *GST* for glutathione S-transferase protein, *MTPB-1* coding for metal tolerance protein B. In stress-associated routes: *HSP70* encoding for heat shock protein 70 and *HSP90* encoding for heat shock protein 90. Those are involved in the carbohydrate metabolism routes: *SDHA* encodes a major catalytic subunit of succinate-ubiquinone oxidoreductase, and the *Lhcb-1* gene encodes chlorophyll-binding protein a/b type 1.

The determination of its differential expression was carried out by quantitative PCR using the Brilliant III Ultra-Fast SYBR Green RT-qPCR Master Mix kit (Agilent, USA). For the PCR reaction 2 μl of RNA was added following the manufacturer's recommended protocol. The reactions were carried out by duplicate (*n* = 2) on the Mx3000P thermal cycler (Agilent, Santa Clara, USA), and if the obtained C_T_ differed more than 5% the reaction was processed again. The temperature profile was 50 °C for 10 min, denaturation at 95 °C for 3 min, 40 ring cycles and extension of 95 °C for 20 s and 55 °C to 62 °C for other 20 s (Table 1). At the end of each run, the melting curves were produced to verify the specific amplification of the reaction, the temperature profile used for this curve was 95 °C for 1 min, 55 °C for 30 s and 95 °C for 40 s. Also, the specificity of the amplification was verified through 1.5% agarose gel stained with GelRed fluorescent (Biotium, Fremont, USA). The expression was quantified by the delta threshold cycle (C_T_) method, using TBP-1 (which encodes for TATA-box-binding protein) as the constitutive gene (Table 1) taking into account a corrected efficiency for all reactions of 1.8^32,33^.

**Table 1:** Information of selected primers.

Name	Direction	Sequence	Size (pb)	Temperature annealing (°C)
*HSP70*	(5′-3′)	TCTTGGCGATGATGGGGTTGC	220	61
	(3′-5′)	GACTCGAAGAACGCCCTGGAG		
*HSP90*	(5′-3′)	CTGCTCCTTGCTGCTTCCCG	211	60
	(3′-5′)	GGGACAGTATGCCAAGTTCTGGA		
*pAPX*	(5′-3′)	AGAGGATCACGGGTCCATGCAC	152	62
	(3′-5′)	GATGCTAAGAGAGGGGCACCAC		
*CAT*	(5′-3′)	GCAAGAACCACAGCCACGCCA	153	62
	(3′-5′)	CCTCGGGCCAGGTCTTGGT		
*GST*	(5′-3′)	CTACAGAGCCACGCCGTCATCG	193	62
	(3′-5′)	CAGCGTGGATCTGGGGTGCT		
*MTPB-1*	(5′-3′)	CGCATGCCATTGGAGAATCACT	219	60
	(3′-5′)	GCAAAATAGCCAGCACCTTCCT		
*SDHA*	(5′-3′)	TGAACCCACTGGATGATGAA	111	55
	(3′-5′)	GGACAAAATTCAGGGGAAGC		
*Lhcb-1*	(5′-3′)	TTCTCCATGTTCGGCTTCTT	227	61
	(3′-5′)	TCCATCAGTCACGACACACA		
*TBP-1*	(5′-3′)	GCAGATATTCTTGATCCCGCTTT	69	60
	(3′-5′)	CGGATGAGGGAACTCAATCTTT		

### Data analysis

For the germination and elongation data, the dose response curve was determined, and the toxicity threshold was calculated, defined as the maximum concentration which there is no significant effect (NOEL). In order to determine the ideality of the curve, the normality of the data was evaluated using the Anderson Darling test and subsequently, a parametric or non-parametric ANOVA was performed depending on the data obtained. Likewise, comparisons were made to assess the differences in germination and elongation between treatments with the Kruskal–Wallis or Dunn´s tests depending on the normality of the data. To evaluate the effect of the interaction between the exposure time and the metal concentration, a general linear model (GLM) was performed, where exposure time and metal concentration were included as factors. The software used for the statistical analysis was GraphPad Prism 5.0 and Minitab 2018.

## Results

### Preliminary determination of germination optimal time and seed viability

**Figure 1:**
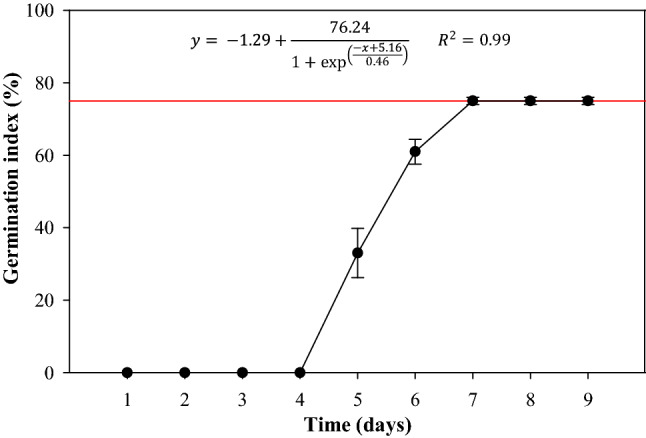
Average germination percentage of *L. perenne* seeds.

The cumulative germination rate of the *L. perenne* seeds is shown in Fig. 1. It follows a typical S or sigmoidal curve behavior. Likewise, it was found that the emergence of the radicular structure started on average on the fourth day, and it was stabilized on day seven with a percentage of 75% without statistically significant changes for the following two days of observation (*P* = 0.405). To determine the optimal time, the last day of exponential growth was considered, this because until this day the growth of mature seeds is presented and with the necessary characteristics to continue growing even under stressful conditions.

The curve found is similar to the one obtained by Bewley et al.^34^ who argues that this type of curve is obtained when the seed sample is not uniform, which causes some seeds to complete germination relatively quickly and others require longer times. These cumulative germination curves are normally sigmoidal, typically occurring when dry, viable seeds begin water absorption and a series of events are triggered that end with successful germination^35–37^. The foregoing reflects that, like many of the biological properties, the germination time of the seeds presents approximately a normal distribution. Likewise, that the germination percentage has stabilized at a value less than 100%, and that after three days there have been no increases, implies that only a fraction of the selected sample was able to germinate under imbibition conditions, for which the remaining fraction of approximately 24% corresponded to unviable seeds.

### *Lolium perenne* germination under Cd and Hg exposure

**Figure 2:**
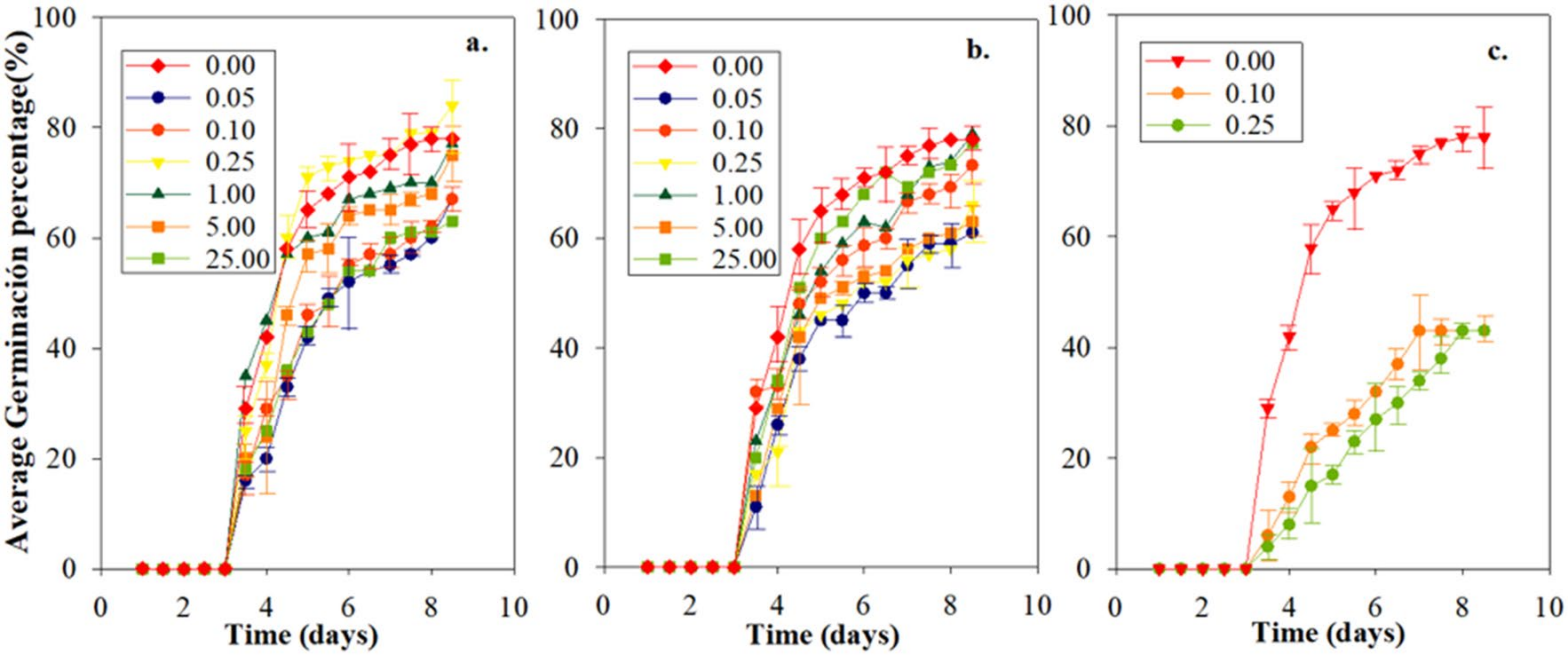
Germination percentages of *L. perenne* exposed to de different metal concentrations. (**a**) Cd^2+^, (**b**) Hg^2+^, (**c**) Cd^2+^–Hg^2+^.

The germination rate was reported daily for nine days, taking two measurements per day (Fig. 2). For the concentrations of Cd^2+^ (Fig. 2a) and Hg^2+^ (Fig. 2b), a germination process like that of Fig. 1 was presented. In contrast, for the binary mixture (Cd^2+^–Hg^2+^) a delay in the stabilization time was observed, as well as a decrease in the percentage of germination on the last day (Fig. 2c).

Although germination started on the same day for all treatments, the number of germinated seeds showed variations between the concentrations for the different metals. For the control seeds, a percentage of germination of 29 ± 2.51% was presented after the first day. In the case of seeds treated with each of the concentrations of the different metals, a decrease in the germination percentage was observed. According to the Kruskal Wallis test, a significant difference with a *P*-value < 0.05 was shown for Cd^2+^, Hg^2+^ and Cd^2+^–Hg^2+^in comparison with the control.

Regarding the results of the Dunn’s test, it was found that in the treatments with Cd^2+^ an inhibition potential was evidenced in the concentrations of 0.05 mg/L (DRM = 28.91), 0.1 mg/L (DRM = 25.55), and 25 mg/L (DRM = 26.27). In the case of Hg^2+^ treatment, the inhibition potential was observed at concentrations of 0.05 mg/L (DRM = 29.27), and 0.25 mg/L (DRM = 27.82) corresponding to the lower germination lines in Fig. 2b. Finally, for the binary mixture, the inhibition potential was observed at both concentrations at 0.10 mg/L (38.57) and 0.25 mg/L (DRM = 38.12).

This potential of heavy metals to decrease germination is due to the fact that germination depends mainly on seed reserves, especially starch, for the supply of metabolites necessary in processes such as respiration, as well as other anabolic reactions that they can be inhibited by metals^38,39^.

Table 2 shows the effect of Cd^2+^, Hg^2+^ and Cd^2+^–Hg^2+^ treatments on different parameters associated with the germination of *L. perenne* seeds such as IGP, FGP, CVG, MGT, and TGI through analysis of variance. It was observed that for the individual metals there was only a significant difference in the IGP for Hg^2+^ with a concentration of 0.05 mg/L and 5.00 mg/L in which there is an inhibition compared to the control. On the other hand, in the case of Cd^2+^–Hg^2+^, statistically significant changes were presented for all the parameters evaluated. This indicates that for the two-mixture concertation (Cd^2+^–Hg^2+^), the inhibition occurred both in the initial and final germination percentage and in the emergency kinetics.

**Table 2:** Germination parameters of *L. perenne* exposed to Cd^2+^ Hg^2+^ and Cd^2+^–Hg^2+^.

Treatment	Concentration (mg/L)	IGP (%)	FGP (%)	CVG (day^−1^)	MGT (day)	TGI
Cd^2+^	0.05	16.00 ± 1.63	67.00 ± 6.81	0.15 ± 0.00	4.91 ± 0.12	0.56 ± 0.05
	0.10	17.00 ± 3.42	67.00 ± 3.79	0.16 ± 0.00	4.58 ± 0.22	0.57 ± 0.04
	0.25	25.00 ± 9.15	84.00 ± 4.32	0.16 ± 0.00	4.67 ± 0.26	0.77 ± 0.07
	1.00	35.00 ± 8.54	77.00 ± 6.61	0.16 ± 0.00	4.59 ± 0.15	0.73 ± 0.07
	5.00	20.00 ± 6.73	75.00 ± 7.90	0.15 ± 0.00	4.93 ± 0.24	0.65 ± 0.07
	25.00	18.00 ± 4.76	63.00 ± 4.76	0.15 ± 0.00	4.81 ± 0.25	0.56 ± 0.01
*P*-value		0.58	0.35	0.90	0.74	0.07
Hg^2+^	0.05	11.00 ± 1.00*	61.00 ± 7.00	0.15 ± 0.01	5.19 ± 0.25	0.53 ± 0.06
	0.10	32.00 ± 1.63	72.00 ± 1.33	0.17 ± 0.01	5.01 ± 0.37	0.64 ± 0.04
	0.25	17.00 ± 6.40	66.00 ± 6.83	0.15 ± 0.01	5.13 ± 0.17	0.57 ± 0.08
	1.00	23.00 ± 3.42	79.00 ± 5.00	0.15 ± 0.00	5.08 ± 0.16	0.70 ± 0.03
	5.00	13.00 ± 5.74*	63.00 ± 3.42	0.15 ± 0.00	4.74 ± 0.33	0.56 ± 0.03
	25.00	20.00 ± 6.32	60.00 ± 11.62	0.17 ± 0.01	4.66 ± 0.26	0.68 ± 0.10
*P*-value		0.02	0.16	0.43	0.21	0.12
Cd^2+^–Hg^2+^	0.10	6.00 ± 1.34*	43.00 ± 1.80*	0.19 ± 0.00*	5.12 ± 0.02*	0.35 ± 0.00*
	0.25	4.00 ± 0.65*	43.00 ± 2.33*	0.17 ± 0.00*	5.72 ± 0.10*	0.32 ± 0.00*
*P*-value		< 0.00	< 0.00	< 0.00	< 0.00	< 0.00
Control	0.00	29.00 ± 2.52	79.00 ± 1.16	0.16 ± 0.01	4.43 ± 0.09	0.74 ± 0.00

The * represents the levels of concentration statistically significant.

Treatments exposed to Cd^2+^ did not present significant differences, which may be due that this metal prevents the absorption and movement of water by the seed^40^. However, this does not occur with exposure to Hg^2+^ that presented significant differences and the results are similar to other studies^41^. These results may be due to the entry of contaminated solution for the seeds exposed to Hg^2+^ and not for those exposed to Cd^2+^, which would indicate the inhibitory results for PGI in the case of Hg^2+^. Instead, in Cd^2+^–Hg^2+^, the most pronounced antagonistic effect was presented, very similar to that reported in other studies that associate it with the interaction between metals resulting from the formation of metal complexes with greater toxicity, or also with the influence it generates, one metal in binary mixtures in the adsorption of the other metal in the solution^38,42^.

### Effects in the root and stem elongation

**Figure 3:**
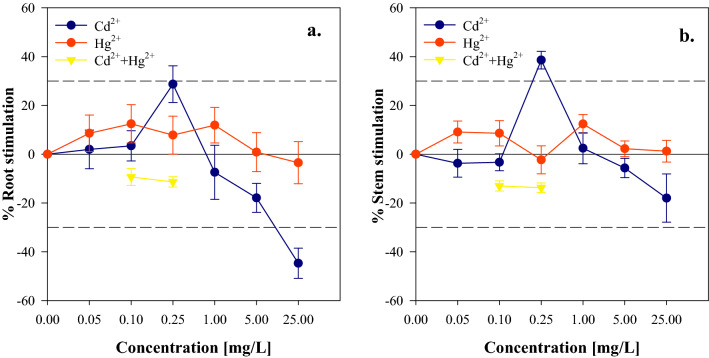
Dose response curve of Cd^2+^ Hg^2+^ and Cd^2+^–Hg^2+^ in elongation of *L. perenne* (**a**) root stimulation (**b**) stem stimulation.

Root and stem elongation of *L. perenne* at different concentrations of Cd^2+^, Hg^2+^ and Cd^2+^–Hg^2+^ were evaluated after the ninth day on which a percentage greater than 80% of germination was reached for all treatments. Regarding the statistical analyses, it was found that for the cases of Cd^2+^ and Cd^2+^–Hg^2+^ there is a statistically significant effect (*P*-value < 0.05) of the concentrations in the elongation of both the root and the stem. For the treatments with Hg^2+^, there was no statistical difference between concentrations, therefore, there was no inhibitory or favorable effect for the seeds treated with this metal (Fig. 3).

Regarding the comparison tests, it was found that the root exposed to Cd^2+^ in concentrations of 0.25, 5 and 25 mg/L (q = 5.77, q = 4.28 and q = 8.85) presented a statistically significant difference. For the concentration of 0.25 mg/L a stimulation of 28.7% was found and for the two remaining concentrations it was observed and inhibitory effect. The root exposed to Cd^2+^–Hg^2+^ presented statistically significant differences for the concentrations of 0.10 and 0.25 mg/L (q = 9.47 and q = 9.52). For the stem exposed to Cd^2+^ the concentration with a statistically significant difference was 0.25 mg/L (q = 6.07) presenting a stimulation of 38.6%, and for the stem exposed to Cd^2+^–Hg^2+^, a statistically significant difference was found for the concentrations of 0.10 and 0.25 mg/L (q = 8.06 and q = 8.39) with an inhibitory effect in both concentrations.

The behavior observed in the case of Cd^2+^ can be based on a hormetic process of dose response, where at low concentrations a stimulus occurs in the plant, and at higher concentrations an adverse effect occurs^43^. These percentages of stimulation found were similar to those reported in multiple toxicology databases^43^. Calabrese and Blain^44^ indicated that the magnitude of the stimulatory response is characterized by being moderate, being in most cases between 30 and 60% above the control values.

Accordingly, the maximum level with no observable effect (NOEL) was that corresponding to the concentration of 0.1 mg/L for the early growth of the root and stem in the treatments with Cd^2+^. The importance of NOEL is because at this dose the stimulations in the metabolism of plant organs begin to appear, allowing a favorable effect on elongation to be achieved at higher doses. This stimulation in the metabolism could be characterized by overcompensation to an interruption in the homeostasis of the organism^45^. This overcompensation may be the result of incentive of cell proliferation, which could be related to the ability of Cd^2+^ to replace the functional metal Zn^2+^, allowing the binding of multiple transcription factors to the regulatory regions of genes^46^. The negative effects at high concentrations presented in Cd^2+^ for this study are very similar to those reported by other authors, because it is considered highly toxic^28,40,42,47–49^, while at low concentrations positive effects similar to other studies were presented^45,46,50,51^. This could explain that plants develop oxidative stress when exposed to heavy metals that can cause cellular damage, due to the accumulation of metal ions that generate disturbances in cellular ionic homeostasis^52^.

Regarding the mixture Cd^2+^–Hg^2+^ the inhibition percentages were between 37.8 and 53.3%, presenting the greatest negative effect for the concentration of 0.25 mg/L. In the treatments of both mixtures, an inhibitory response increased with increasing concentration and was greater for the root structure which can be attributed to the mobility of metals from roots to stem and direct contact with the root surface. This shows that in *L. perenne* as in other plants, heavy metals tend to be retained in the root tissues, therefore, the mixing effects are generally greater in the roots than in the stem^21,53^. On the other hand, in the binary mixture the most pronounced antagonistic effect was presented, which may be a consequence of the interaction between the metals that results in the formation of metal complexes with greater toxicity or also the influence that a metal generates in the binary mixtures in the absorption of the other metal in the solution^38,42^.

In the case of Hg^2+^, the absorption could produce serious damage to plants by affecting chlorophyll synthesis and reducing photosynthesis as a result of the replacement of Mg^2+^ by Hg^2+^ ^54^. However, in the present study it was found that there was no inhibitory or stimulating response for any of the Hg^2+^ concentrations^55^. This because the absence of an inhibitory effect has been proven on growth of young tissue, which is little affected by increasing concentrations of Hg^2+^. Similarly, it has been found that young plant tissue, which is still in the growth stage, has the possibility of internally diluting Hg^2+^ concentrations by increasing biomass^56,57^.

### Changes in gene expression of *L. perenne*

**Figure 4:**
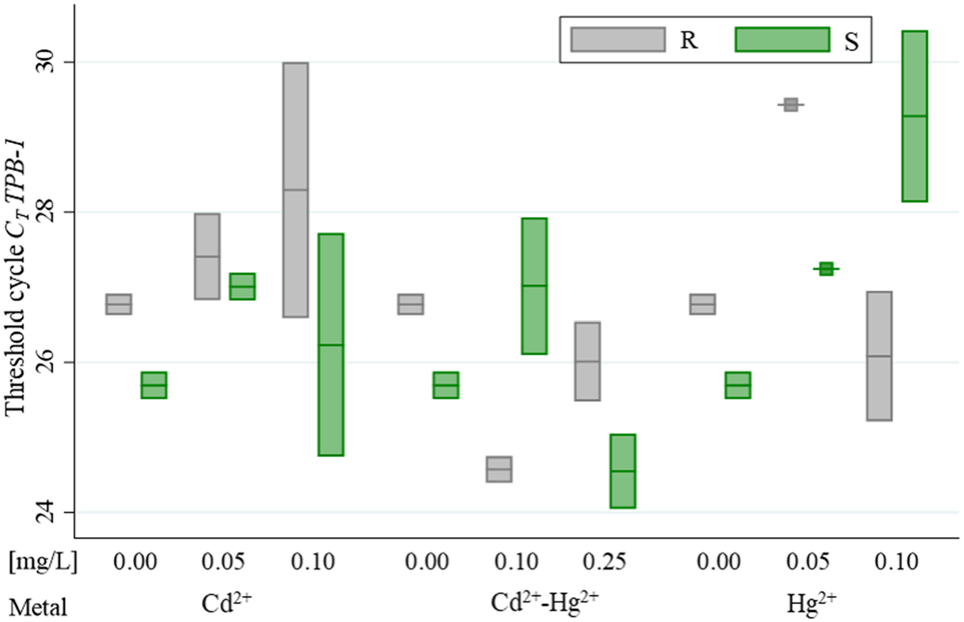
Mean of *C*_*T*_ for the gen *TBP-1* of *L. perenne* exposed to de different treatments (R: root and S: stem).

The evaluation of the genetic expression was performed by RT-qPCR, nevertheless, the specificity of the amplification was corroborated by melting curve and agarose electrophoresis. For the relative quantification the constitutive gen selected was TBP-1, in order to corroborate the viability of this selection, the C_T_ variation among the treatments was evaluated by analysis of variance using the general linear model finding not differences neither roots nor stems among the treatments (Fig. 4) (*P* value > 0.05 for the metals, plant structure, and concentrations), which indicates that this gene is suitable to be used as a gene for reference in relative quantification.

### Differential *L. perenne* gene expression in response to different treatments

**Figure 5:**
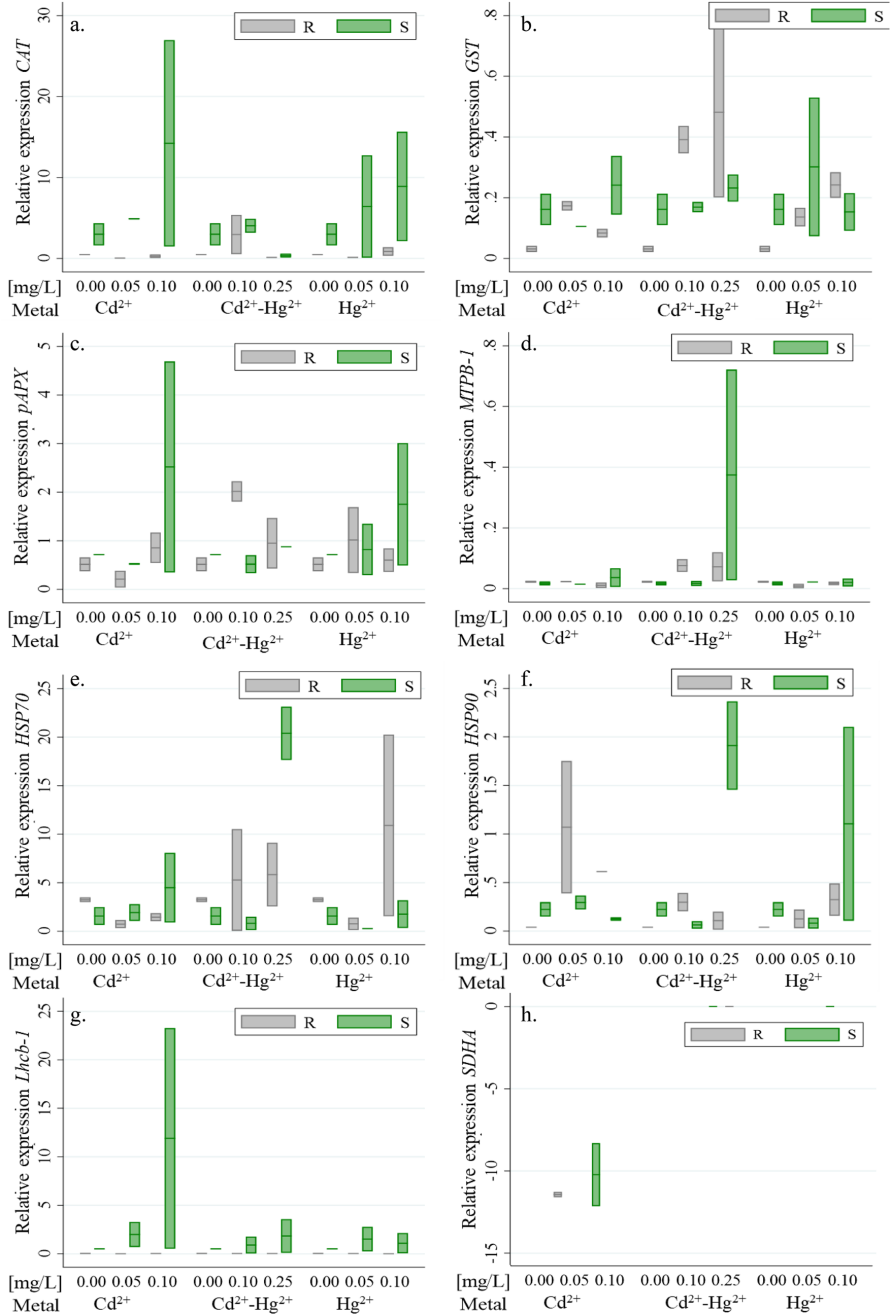
mean of gen relative expression in roots and stems expose for the different treatments (**a**) *CAT*, (**b**) *GST*, (**c**) *pAPX*, (**d**) *MTPB-1*, (**e**) *HSP70*, (**f**) *HSP90*, (**g**) *SDHA*, (**h**) *Lhcb-1.* (R: root and S: stem).

The relative expression of genes encoding for different categorical routes was evaluated for roots and stems, the magnitude of this expression changes (fold) it is shown in Fig. 5. For the detoxification response, the gen *CAT* (Fig. 5a) was upregulated in the stems for the three different treatments having the highest relative expression when the plant was contaminated with the Hg^2+^, in contrast to the root which has a moderated expression being even less than the reference gene in some treatments. This response is the result that the *CAT* activity has been associated with H_2_O_2_ removal and antioxidant protection in peroxisomes and organelle glyoxysomes found in the entire plant, the enzyme catalase being one of the first to be expressed when oxidative degradation occurs^58^. In contrast, *pAPX* (Fig. 5c) was overexpressed in the roots exposed to Cd^2+^ at the higher concentration, and for the stems and roots exposed to Hg^2+^ but not for Cd^2+^ + Hg^2+^. This result is similar to the ones obtain in Alfalfa and maize roots where *pAPX* responded by increasing activity at a moderate degree of toxicity^59^.

The *GST* gene (Fig. 5b) was downregulated in roots and stems exposed to the different treatments, especially in the different concentrations with Cd^2+^. However, it was evident that the treatments have an influence on the gene expression in comparison with the negative control. GST enzymes catalyze the conjugation between various xenobiotics with electrophilic centers, such as those produced by the metal ions of Cd^2+^ and Hg^2+^, and the nucleophile glutathione (GSH), thus marking the xenobiotic for vacuolar sequestration^60,61^. The resulting GSH conjugates are generally less toxic and more soluble in water than the original xenobiotics^62,63^.

In the category stress-related genes, the higher expression levels were for the *HSP70* (Fig. 5e), it was upregulated in stems especially exposed with Cd^2+^–Hg^2+^ and Hg^2+^ at the higher concentrations, however, the expression in the roots did not show a dose response behavior. Hsp70 proteins are crucial as molecular chaperones that inflict the catalysis necessary to rapidly recruit and release proteins^27,64^. This protein has been found to play an important role in the programmed cell death of the leaves of the species *Aponogeton madagascariensis*, this in order to achieve the elimination of cells compromised with stress. The *Hsp90* (Fig. 5f) was only overexpressed in roots exposed with Cd^+2^ and the stems exposed with Cd^2+^–Hg^2+^ and Hg^2+^. Hsp90 proteins are responsible for safeguarding the integrity of the cell membrane when the plant organism is exposed to heavy metals, resulting in reductions in the germination rate and root length^65^. Finally, for the category related to energy production, *Lhcb-1* (Fig. 5h) was only expressed in the stem as expected, and it was upregulated in the three treatments, nonetheless, the response was lower for the Cd^2+^–Hg^2+^. The gens *MTPB-1* for detoxification (Fig. 5d) and *SDHA* (Fig. 5g) energy production category were not express either for the control and the different treatments. Since, just two replicates of each treatment could be made, the statistical comparisons were not performed. However, the changes expression levels observed for the genes *CAT*, *GST*, *pAPX*, *HSP70* and *HSP90* (Fig. 5) indicated physiological mechanisms used by the plant to repair the damage conferring tolerance to grow in contaminated medium with heavy metals and even enhancing their growth under concentrations that are minimal to inhibitory, being the hormetic growth evident. These results are in accordance with previous works, where it has been found that *L. perenne* is a phytoremediator plant of this type of metals^21^ due to its accumulative capacity, and with the evaluation of these genes it is shown that *L. perenne* can be used in soils for the removal of contaminants such as Hg and Cd.

The RT-qPCR analysis showed that the relative expression levels in the root and stem of *L. perenne* for the *MTPB-1* and *Lhcb-1* genes did not present statistical difference between the different treatments. While for the rest of the genes (*CAT, pAPX, GST, HSP70* and *HSP90*) there was a difference in the relative expression for the concentration factor, however, for the metal exposure factor there is no statistically significant difference. In relation to the changes in the relative expression of each gene with respect to the different concentration levels, the following was observed: for the *CAT* gene there was a decrease in the root and stem for a concentration of 0.25 mg/L, the *GST* gene increased in the root for all concentrations and in the stem for a concentration of 0.10 mg/L, *pAPX* increased in the concentration of 0.10 mg/L in the stem, while *HSP90* increased in the concentration of 0.10 mg/L in the root, and finally *HSP70* increased in the concentration of 0.25 mg/L in the stem (Table 3).

**Table 3:** Significantly different concentration levels for the expression of each gene in the root and stem structures of *L. perenne.*

	Root			Stem	
	Concentration [mg/L]	*P*-value		Concentration [mg/L]	*P*-value
*CAT*	0.05–0.10	0.037	*CAT*	0.05–0.10	0.019
*GST*	0.05–0.10–0.25	0.000	*pAPX*	0.10	0.009
*HSP90*	0.10	0.015	*GST*	0.05–0.10–0.25	0.020
			*HSP70*	0.25	0.047

## Conclusions

The results of this study indicated that *L. perenne* is a plant with a significant phytoremediator capacity for heavy metals as Cd^2+^ and Hg^2+^ individually, since the effects at the morphological and molecular levels indicating the presence of tolerance mechanisms observed in the germination and elongation of the roots and stems. However, for the treatment Cd^2+^–Hg^2+^, the significant inhibition in the germination and elongation were observed and low levels of gen expression, suggesting that the phytoremediation potential may be affected if metal mixtures are found in the medium. Regarding the morphological response of the individual metals, it was found that germination presented an inhibition response relative to increasing concentration. Nevertheless, the plant grows, and an important germination percentage was obtained.

The NOEL concentration for Cd^2+^ was 0.10 mg/L, in this concentration the longitude increases in both roots and stems, and the change of the genetic expression was observed. This could indicate this level of exposure is where the molecular tolerance response occurs in which the plant seeks to counteract the stress caused by metals mainly performed by detoxification mechanisms, which would be an explanation for the increase in morphological response at the subsequent concentration of 0.25 mg/L. Even though the selection of concentrations occurred as a result of the dose response curve found for Cd^2+^, the molecular response was similar for this metal and for Hg^2+^. Indicating the detoxication molecular rout for this metal has a different morphological response.

Although the limited number of biological replicas did not allow made statistical comparisons among levels of gen expression, changes were observed, these results being a starting point for further investigation of the molecular mechanisms of phytoremediation plants, and their potential use.
